# Survival and Extent of Breast Surgery in Patients with Complete Response After Neoadjuvant Therapy: A Retrospective Analysis from the EUSOMA Database

**DOI:** 10.3390/cancers18213536

**Published:** 2026-11-03

**Authors:** Federica Martorana, Damiano Gentile, Mariano Tomatis, Antonio Ponti, Lorenza Marotti, Cynthia Aristei, Nicolò Matteo Luca Battisti, Kwok Leung Cheung, Olaf Johan Hartmann, Isabel T. Rubio, Donatella Santini, Francesco Sardanelli, Elzbieta Senkus, Peter van Dam, Giuseppe Curigliano, Giuseppe Catanuto, on behalf of the EUSOMA Working Group

**Affiliations:** 1 Department of Clinical and Experimental Medicine, University of Catania, 95123 Catania, Italy; federica.martorana@humanitascatania.it; 2 Humanitas-Istituto Clinico Catanese, Misterbianco, 95045 Catania, Italy; 3 Department of Biomedical Sciences, Humanitas University, Rozzano, 20089 Milan, Italy; 4 Breast Unit, IRCCS Humanitas Research Hospital, Rozzano, 20089 Milan, Italy; 5 European Society of Breast Cancer Specialists (EUSOMA), 50019 Florence, Italy; mariano.tomatis@eusoma.org (M.T.); antonio.ponti@eusoma.org (A.P.); lorenza.marotti@eusoma.org (L.M.); 6 CPO Piemonte, 10123 Turin, Italy; 7 Radiation Oncology Section, Department of Medicine and Surgery, University of Perugia, 06156 Perugia, Italy; cynthia.aristei@unipg.it; 8 Perugia General Hospital, Sant’Andrea delle Fratte, 06156 Perugia, Italy; 9 Breast Unit, Department of Medicine, The Royal Marsden NHS Foundation Trust, London SW3 6JJ, UK; nicolo.battisti@rmh.nhs.uk; 10 Academic Unit for Translational Medical Sciences, School of Medicine, University of Nottingham, Royal Derby Hospital Centre, Nottingham NG7 2UH, UK; kl.cheung@nottingham.ac.uk; 11 Akershus University Hospital, 1478 Nordbyhagen, Norway; olaf.johan.hartmann@ahus.no; 12 Breast Surgical Oncology, Clinica Universidad de Navarra, Cancer Center Universidad de Navarra, 28027 Madrid, Spain; irubior@unav.es; 13 Department of Pathology, IRCCS Policilnico S. Orsola, University of Bologna, 40138 Bologna, Italy; donatella.santini.ab@gmail.com; 14 Lega Italiana per la Lotta Contro i Tumori (LILT) Milano Monza Brianza, 20133 Milan, Italy; francesco.sardanelli@legatumori.mi.it; 15 Department of Oncology and Radiotherapy, Medical University of Gdańsk, 80-211 Gdańsk, Poland; 16 Breast Unit, University Clinical Center, 80-952 Gdańsk, Poland; 17 Multidisciplinary Oncologic Center, Antwerp University Hospital, 2650 Edegem, Belgium; peter.vandam@uza.be; 18 Department of Oncology and Hemato-Oncology, University of Milano, 20122 Milan, Italy; giuseppe.curigliano@ieo.it; 19 Division of New Drugs and Early Drug Development for Innovative Therapies, European Institute of Oncology, IRCCS, 20141 Milan, Italy

**Keywords:** breast cancer, breast-conserving surgery, mastectomy, neoadjuvant therapy

## Abstract

Background: Optimal management of breast cancer (BC) patients with pathological complete response (pCR) after neoadjuvant therapy (NAT) remains debated. We performed a retrospective analysis to evaluate whether the extent of surgery influences survival outcomes in this patient population. Methods: Using the EUSOMA database, we retrospectively collected data of women with pCR after NAT, excluding those with BRCA1/2 mutations. The cohort was stratified by surgery type, i.e., breast-conserving surgery (BCS) or mastectomy. Relapse-free survival (RFS) and overall survival (OS) were compared using Cox regression. Results: Of 467 patients, 327 (70.0%) underwent BCS and 140 (30.0%) underwent mastectomy. Women treated with mastectomy were more likely to have multifocal tumors and advanced disease. After a median follow-up of 5.6 years, 7.3% of patients experienced BC recurrence. Axillary nodal involvement (cN+) and mastectomy were independently associated with recurrence, occurring in 11.4% of cN+ compared to 3.7% of cN0 patients (HR 3.02; 95% CI: 1.40–6.51; *p* = 0.005) and in 12.5% of mastectomy vs. 5.2% of BCS patients (HR 2.08; 95% CI: 1.05–4.11; *p* 0.035). cN+ patients also showed significantly higher mortality rates (9.4% vs. 1.6% of cN0; HR 6.16; 95%CI: 2.09–18.17; *p* = 0.001). However, surgery type did not impact OS. Conclusions: In this cohort of BC patients with pCR after NAT, BCS was not associated with a higher risk of recurrence compared with mastectomy. Baseline nodal involvement independently predicted recurrence and mortality; however, the type of surgery did not impact OS. These findings support BCS as a safe treatment option for this patient population.

## Simple Summary

As the number of breast cancer patients achieving pathological complete response after neoadjuvant therapy increases, the choice regarding breast surgery extent becomes pivotal. This analysis from the EUSOMA database shows that breast-conserving surgery provides survival outcomes comparable to mastectomy in patients with pathological complete response. While axillary nodal involvement remains correlated with worse prognosis, mastectomy does not seem to improve survival. These findings support the efforts toward surgical de-escalation and reinforce breast-conserving surgery as a safe, evidence-based option for selected patients. In this context, current clinical practice should incorporate shared decision-making and avoid surgical overtreatment whenever feasible.

## 1. Introduction

Neoadjuvant therapy (NAT) is routinely used in breast cancer (BC) patients, particularly in selected early-stage subtypes. Initially aiming to downstage inoperable tumors and facilitate surgical resection [1], NAT has evolved into a powerful tool with broader clinical utility. Indeed, NAT allows assessment of in vivo tumor chemosensitivity, enabling tailored post-operative treatment strategies [2,3]. The extent of response, especially the achievement of a pathologic complete response (pCR), is a reliable surrogate marker for long-term oncologic outcomes, including recurrence-free survival (RFS) and overall survival (OS) [4,5,6]. Still, the opportunity to reduce surgery extent remains a meaningful advantage of tumor shrinkage following NAT. Indeed, NAT can make patients suitable for breast-conserving surgery (BCS) even if initially considered for mastectomy [7,8,9,10]. In the upfront surgical setting, several studies have demonstrated that BCS combined with radiotherapy achieves equivalent, or in some cases superior, outcomes to mastectomy, both in terms of survival and recurrence rates [11,12,13]. Moreover, BCS is associated with favorable cosmetic results and a positive impact on quality of life [14,15]. In the context of NAT, growing evidence indicates that, compared to mastectomy, BCS remains safe for appropriately selected patients [16,17,18]. However, the transition from a planned mastectomy to BCS in patients who favorably respond to NAT remains inconsistent [19]. Concerns persist about the potential risk of higher local recurrence following BCS after NAT, fueled in part by data from older randomized trials [20,21]. To address this research gap, we evaluated characteristics and oncologic outcomes of BC patients treated with NAT who achieved pCR and underwent BCS or mastectomy at EUSOMA-certified centers, in order to compare the RFS and OS between the two surgical groups, identify clinical and pathological factors associated with recurrence and mortality, and assess whether the type of surgery remained an independent predictor of outcome after adjusting for baseline disease stage and tumor biology.

## 2. Materials and Methods

The EUSOMA database is a centralized, prospectively maintained repository of pseudonymized patient-level data on primary BC, contributed by European breast centers that pursue EUSOMA certification and consent to participate in benchmarking and research efforts. The database contains no personally identifiable information. We extracted and analyzed data from 49 breast centers. We included female patients diagnosed with non-metastatic BC who received NAT, including chemotherapy with or without targeted agents but not hormonal therapy, and underwent surgery between 2016 and 2019, with a minimum follow-up after surgery of 5 years. Only patients with pCR, defined as absence of residual invasive disease in the breast and in axillary lymph nodes at the final pathology report, were eligible [4]. We excluded women with known germline pathogenic variants in BRCA1/2, which constitutes a well-established indication for bilateral prophylactic mastectomy. The following variables were registered: surgery year, surgery type (BCS or mastectomy), age, focality (single, multiple), clinical stage of primary (cT), clinical nodal status (cN), TNM stage (according to the AJCC staging system, 7th edition), immunophenotype of disease [i.e., hormone receptor positive (HR+)/HER2 negative (HER2-); HR+/HER2 positive (HER2+), HER2+, triple negative (TNBC)], grade, Ki67 proliferation index, adjuvant radiotherapy, and adjuvant endocrine therapy (ET). All the analyses were conducted by stratifying the study population into two groups according to the type of surgery: BCS or mastectomy. For descriptive statistics, we used median and interquartile ranges (IQRs) to report continuous variables and proportions for categorical variables. We evaluated the incidence of local and distant recurrence and of mortality in the two groups, using univariable Cox regression and including the following variables: surgery type, age, tumor focality, baseline tumor staging (cT and cN), molecular subtype, radiotherapy, and endocrine therapy. Variables showing a significant correlation were then included in a stepwise multivariable regression model. The statistical significance level was set at < 0.05. All the analyses were performed using R software version 4.3.2.

## 3. Results

### 3.1. Differences in Patient and Tumor Characteristics

Overall, 1686 BC patients without known germline BRCA1/2 pathogenic mutation included in the EUSOMA database underwent NAT between 2016 and 2019, with 467 (27.7%) achieving pCR. Of these, 327 (70.0%) underwent BCS, while 140 (30.0%) underwent mastectomy. The distribution of surgical procedures remained relatively stable across the study period, with no significant differences between years (*p* = 0.516). The median age of the overall cohort was 51 years (IQR: 43–58), with patients undergoing mastectomy being significantly younger than those treated with BCS (median 49 vs. 51 years; *p* = 0.031). Multifocal tumors were more common among patients undergoing mastectomy (38.6% vs. 12.5%), while single-focal tumors were predominant in the BCS group (87.5% vs. 61.4%, *p* < 0.001). Clinical tumor stage at diagnosis significantly differed between groups, with mastectomy more frequently performed in patients with advanced clinical T stage (cT3–cT4: 27.9% vs. 8.6%; *p* < 0.001) and clinical node-positive disease (cN+: 60.0% vs. 42.8%, *p* = 0.001). Consequently, patients undergoing mastectomy more often had stage III disease (32.9% vs. 9.2%, *p* < 0.001). According to the analysis of molecular variants, patients with TNBC were more frequently treated with BCS (35.2% vs. 20.7%) (*p* = 0.012). Regarding treatment patterns, post-operative radiotherapy was significantly more common in the BCS group (92.0% vs. 48.6%, *p* < 0.001). Patient characteristics are summarized in Table 1.

### 3.2. Recurrence-Free Survival

**Figure 1:**
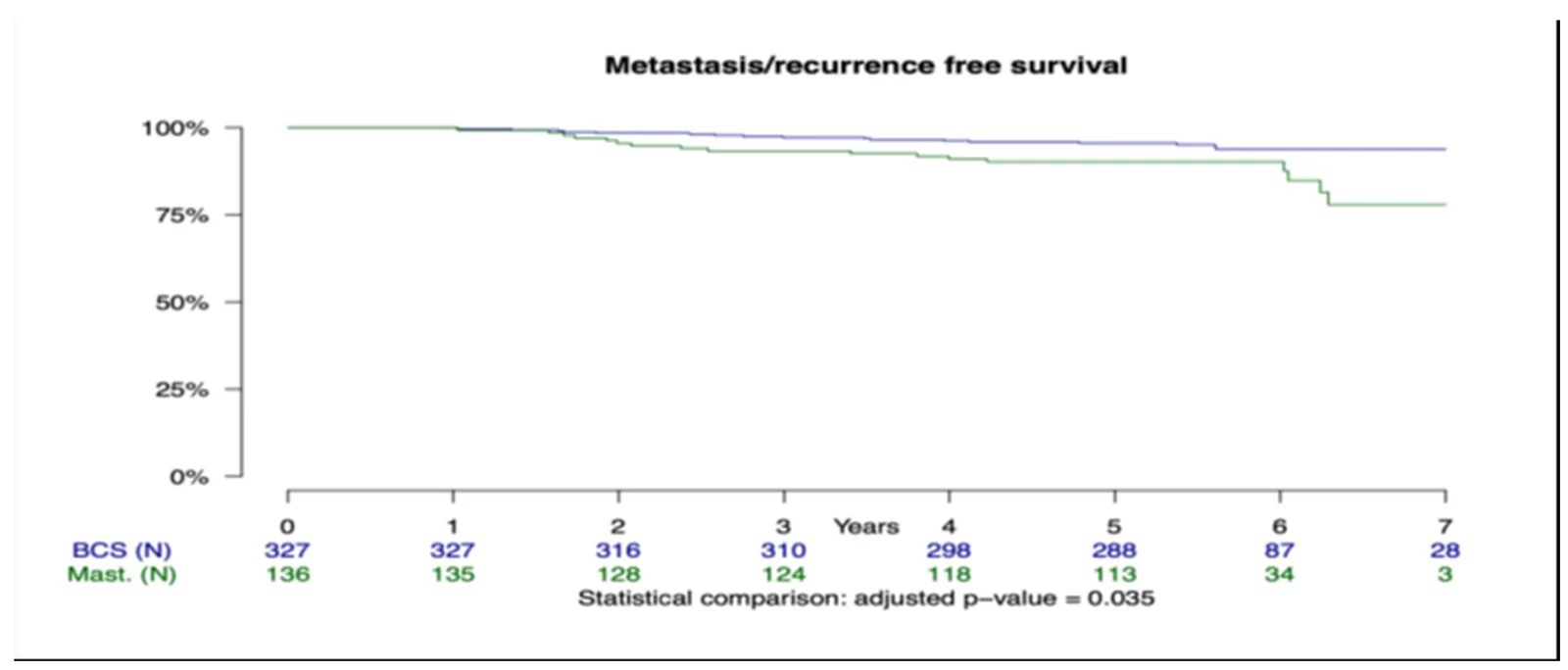
Kaplan–Meier curve for recurrence-free survival according to the type of surgery.

The overall recurrence rate (including loco-regional and distant metastases) in the cohort was 7.3%, with 34 events recorded among 463 patients after a median follow-up of 5.6 years. Recurrence occurred more frequently in patients who underwent mastectomy (12.5%, 17/136) compared to those treated with BCS (5.2%, 17/327). At univariable Cox regression, clinical nodal positivity (cN+) was significantly associated with higher recurrence risk (HR: 3.31, 95% CI: 1.55–7.10, *p* = 0.002), as was the type of surgery, with mastectomy associated with a higher recurrence rate compared to BCS (HR: 2.48, 95% CI: 1.27–4.86, *p* = 0.008). Clinical T4 tumors also showed increased recurrence risk (HR: 4.44, 95% CI: 1.19–16.56, *p* = 0.026). Other variables, including age, tumor focality, BC subtype, use of post-operative radiotherapy, and ET, were not significantly associated with recurrence risk. In the multivariate Cox regression model, both cN+ status (HR: 3.02, 95% CI: 1.40–6.51, *p* = 0.005) and surgical approach (HR for mastectomy vs. BCS: 2.08, 95% CI: 1.05–4.11, *p* = 0.035) remained independently associated with recurrence. Clinical nodal involvement at baseline emerged as the strongest predictor of recurrence (Table 2). Figure 1 shows a significantly superior RFS in the BCS group compared to the mastectomy group (log-rank *p* = 0.003).

### 3.3. Overall Survival

**Figure 2:**
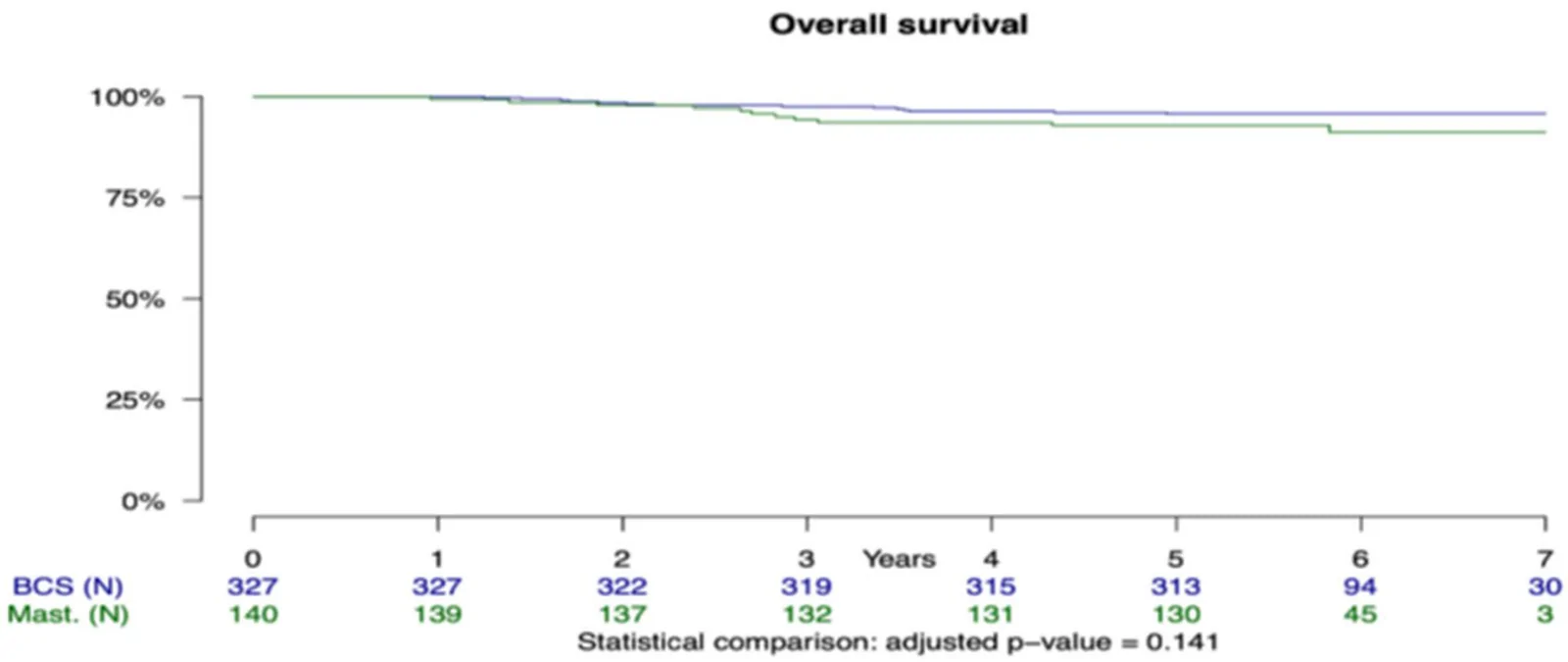
Kaplan–Meier curve for overall survival according to the type of surgery.

The overall mortality rate for the cohort was 5.4%, with 25 events recorded among 467 patients after a median follow-up of 5.6 years. Mortality was higher in the mastectomy group (7.9%, 11/140) compared to the BCS group (4.3%, 14/327). In univariable Cox regression analysis, baseline nodal involvement (cN+) was significantly associated with increased mortality risk (HR: 5.93, 95% CI: 2.04–17.29, *p* = 0.001). Tumor size (cT3) was associated with a non-statistically significant trend toward higher mortality (HR: 3.16, 95% CI: 0.91–10.91, *p* = 0.069). Other variables, including age, tumor focality, BC subtype, surgical type, adjuvant radiotherapy, and ET, were not significantly associated with OS in univariable models. In the multivariate Cox regression model, cN+ status remained an independent and strong predictor of mortality (HR: 6.01, 95% CI: 1.94–18.60, *p* = 0.002), and this was confirmed in the stepwise regression model (HR: 6.16, 95% CI: 2.09–18.17, *p* = 0.001). No other variable, including the type of surgery or the administration of ET, reached statistical significance in multivariate analysis (Table 3). These results are represented in Figure 2, which showed no statistically significant difference in OS between BCS and mastectomy (log-rank *p* = 0.191).

## 4. Discussion

Appropriate surgical approach for BC patients treated with NAT who achieve pCR is still debated, and in many cases, the decision between BCS and mastectomy often lacks clear guidance [19]. Mastectomy is still performed in a significant proportion of patients achieving pCR, potentially due to concerns regarding oncological safety of BCS [22,23]. However, if mastectomy does not confer superior survival outcomes compared to BCS, its physical and psychological burdens cannot be justified [24,25,26]. Our study addresses these uncertainties by comparing oncologic outcomes between BCS and mastectomy in BC patients achieving pCR after NAT.

Despite substantial advances in systemic therapies, imaging, and surgical techniques, uncertainties persist about comparing outcomes of BCS vs. mastectomy. Recent large-scale meta-analyses have produced conflicting results that further complicate surgical decision-making in this setting. For example, the Early BC Trialists’ Collaborative Group (EBCTCG) meta-analysis included 4756 women from ten randomized trials comparing NAT with adjuvant chemotherapy [20]. Despite NAT increasing the chances of BCS (65% in the NAT group vs. 49% in the adjuvant group, *p* < 0.001), it also showed a higher 15-year local recurrence rate (21% vs. 16%; *p* < 0.001). Importantly, there were no significant differences in the rates of distant recurrence (38.2% vs. 38.0%; rate ratio: 1.02, 95% CI: 0.92–1.14, *p* = 0.66) or BC-specific mortality (34.4% vs. 33.7%; rate ratio: 1.06, 95% CI: 0.95–1.18, *p* = 0.310). However, these results were confounded by outdated treatment regimens, incomplete radiotherapy data, and variability in local therapy. Another meta-analysis by Qin et al. [21] analyzed 5018 patients, comparing outcomes between 2120 undergoing BCS and 2898 undergoing mastectomy after NAT. This study reported superior five-year OS (OR: 2.68, 95% CI: 2.19–3.28, *p* < 0.001) and RFS (OR: 3.11, 95% CI: 1.80–5.38, *p* < 0.001) in the mastectomy group. However, the conclusions were limited by heterogeneity in patient populations, variability in treatment protocols, and a lack of randomization, which introduced potential selection bias.

Despite these uncertainties, pCR remains a robust surrogate marker for superior prognosis in BC patients. In various studies, pCR has been consistently associated with improved RFS, distant-RFS (DRFS), and OS, compared to non-pCR. In the EORTC 10994/BIG 1-00 trial [27], involving 1289 patients, pCR was observed in 18% of cases. These patients had a 60% reduced risk of recurrence compared to non-pCR patients (HR: 0.40, 95% CI: 0.25–0.64, *p* < 0.001). Similarly, the CTNeoBC pooled analysis [2], which included data from 12 neoadjuvant trials and over 11,000 BC patients, proved the prognostic value of pCR. Patients achieving pCR had significantly lower HRs for RFS (HR: 0.44, 95% CI: 0.39–0.51) and OS (HR: 0.36, 95% CI: 0.30–0.44). In a retrospective cohort study by Chou et al. [28], 1047 BC patients treated with NAT were included, and the overall pCR rate was 22.2%. Patients achieving pCR had significantly better 5-year locoregional RFS (94.1% vs. 87.9%, *p* = 0.004). Notably, comparison between patients who underwent BCS and those who underwent mastectomy failed to show any significant difference in the overall locoregional recurrence rate of the two groups, 8.8% in the BCS group vs. 10.7% in the mastectomy group (*p* = 0.303). Lastly, the study by Gentile et al. [5], conducted on 495 patients undergoing NAT, reported a pCR rate of 29.9%. The pCR group had an improved 5-year RFS (94.9% vs. 70.5%, *p* < 0.001), DRFS (93.3% vs. 71.5%, *p* < 0.001), and OS (98.2% vs. 79.6%, *p* < 0.001) compared with the non-pCR group.

Building on the evidence that NAT downstages tumors and increases the rate of BCS, an important consideration is the oncologic safety of BCS compared to mastectomy. In our study, BCS was not associated with an inferior recurrence rate compared to mastectomy, even after multivariable adjustment. Taking into account the inherent limitations of a retrospective analysis, our results suggest that BCS is as safe as mastectomy in selected patients achieving pCR after NAT.

Our findings are consistent with previous evidence. A Dutch cohort of 561 patients treated with NAT reported no significant differences in RFS or OS between BCS and mastectomy, even after multivariable analysis adjusting for clinical tumor and nodal status, molecular subtype, and pCR, with adjusted HR of 1.31 (95% CI: 0.81–2.13) [29]. Notably, the study highlighted that achieving a breast pCR was independently associated with an improved RFS (HR: 0.467; 95% CI: 0.238–0.918; *p* = 0.027), which again reflected the prognostic value of the response to NAT. A secondary analysis of the I-SPY2 trial [30], which included 1462 patients, further corroborated these findings. The study demonstrated that locoregional RFS, RFS, and OS were not significantly associated with the type of surgery after NAT. A retrospective study of 730 patients from China found no significant differences in 5-year locoregional recurrence (6.9% vs. 7.6%; *p* = 0.805) or RFS (83.4% vs. 78.2%; *p* = 0.514) between BCS and mastectomy groups [31]. Importantly, the BCS group had better BC-specific survival (HR: 0.27; *p* = 0.025) and OS (HR: 0.25; *p* = 0.018), suggesting potential quality-of-life benefits without compromising oncologic safety. A meta-analysis by Sun et al. [17] involving 16 studies and 3531 patients revealed that BCS after NAT was linked to a 49% lower risk of distant recurrence, with improvements in both RFS and OS (*p* < 0.01). Similarly, the New Jersey State Cancer Registry study by Arlow et al. [18] showed that BCS plus radiotherapy resulted in better 10-year BC-specific survival compared to mastectomy alone (*p* = 0.0046). Other large-scale studies, such as those by Nobrega et al. [32] and Gwark et al. [33], found that BCS with adjuvant radiotherapy resulted in superior OS and RFS. Additionally, Krug et al. [34] reported that BCS was associated with significantly improved RFS and OS in patients with early-stage triple-negative BC after NAT.

These findings also raise the question of whether local disease control and distant recurrence contribute differently to long-term survival after BCS plus radiotherapy and mastectomy. Mokbel [35] proposed that preferential homing of reactivated circulating and disseminated tumor cells to the preserved breast, potentially mediated by the CXCR4–SDF-1 axis, could provide a biological explanation for the coexistence of higher locoregional recurrence and improved OS reported in some comparisons of breast-conserving therapy with mastectomy. This remains a hypothesis rather than an established mechanism. In our cohort, RFS combined local, regional, and distant recurrences, precluding assessment of these patterns separately. Longer follow-up with site-specific recurrence data would help determine whether differences in distant recurrence emerge over time and subsequently affect OS in patients achieving pCR after NAT. The clinical interpretation of these findings must also account for the timing of pCR assessment. As pCR can only be definitively established by pathological examination of the surgical specimen, our results provide reassurance regarding outcomes in patients subsequently confirmed to have achieved pCR, rather than a preoperative criterion for choosing BCS over mastectomy. This distinction was also emphasized in our recent publication [36], which identified reliable preoperative assessment of complete response through imaging, image-guided biopsy, and improved response-assessment strategies as a priority for surgical de-escalation. Until such approaches are sufficiently validated, surgical planning should remain based on the initial disease extent, clinical and imaging findings after NAT, the feasibility of achieving clear margins, and patient preferences.

Taken together, our results support the conclusion that BCS does not lead to inferior oncologic outcomes compared to mastectomy in patients achieving pCR after NAT. These findings suggest that other factors, particularly baseline clinical nodal positivity, which emerged as the strongest predictor of both recurrence and mortality in our analysis, should be considered primary drivers of prognosis rather than the extent of breast surgery itself.

Of note, it should be taken into account that pCR is always defined “post hoc”. The multidisciplinary team cannot base the choice between BCS and mastectomy on this information. Thus, we can speculate that, when pCR is expected, BCS should always be considered.

This study has several limitations. First, its retrospective nature coupled with the small number of events registered limits the strength of the conclusions and makes the findings hypothesis-generating. With only 34 recurrences and 25 deaths, stepwise variable selection may have produced unstable or overfitted models and left residual confounding by baseline characteristics not retained in the final models. Selection biases cannot be excluded, particularly given the absence of standardized criteria for surgical decision-making following NAT. For example, tumor stage heterogeneity within categories, such as T2 tumors, introduces potentially relevant prognostic differences. Larger tumors within the same category not only carry a poorer long-term prognosis, with higher risks of recurrence and inferior survival, but are also more likely to be treated with mastectomy compared with smaller tumors. The same bias is introduced by the lack of a precise definition of clinical nodal involvement (i.e., knowing whether a patient has cN1 or higher disease), especially with regard to the choice of post-operative RT. Consequently, the interplay between baseline tumor characteristics, surgical choice, and long-term clinical outcomes could not be adequately captured in this analysis. Moreover, we could not assess how radiological downstaging, tumor size distribution, or patient preferences may have influenced surgical planning. This limitation is especially relevant in a setting where oncologic outcomes are similar, and patient-centered factors often guide surgical decisions. Additionally, variables related to surgeon expertise or access to advanced oncoplastic techniques were not captured. It is plausible that limited availability or confidence in performing complex BCS procedures may have contributed to a higher mastectomy rate. Another limitation relates to the absence of detailed data on radiotherapy planning and delivery, including boost administration or coverage of regional nodal basins, which could have influenced recurrence patterns and outcomes. Furthermore, while the EUSOMA certification promotes a high standard of care across centers, we were unable to explore potential inter-institutional or geographical variations in surgical practice, as country-specific analyses were not permitted under data protection policies. Nevertheless, the inclusion of a large, multi-national cohort from EUSOMA-certified centers, which operate under harmonized quality standards, helps to mitigate concerns regarding heterogeneity in care delivery and strengthens the generalizability of our findings.

## 5. Conclusions

In conclusion, this study provides observational evidence supporting BCS in appropriately selected patients, while encouraging careful consideration of the need for more extensive surgery in this setting. Clinical factors such as baseline nodal status should be considered alongside surgical extent when estimating prognosis. By addressing persistent misconceptions and aligning surgical strategy with individualized risk, this study supports shared decision-making aimed at minimizing overtreatment and avoiding unnecessary mastectomies.

## Figures and Tables

**Table 1:** Population’s characteristics.

Variable		Total 467 (100%)	BCS 327 (70.0%)	Mastectomy 140 (30.0%)	*p*-Value
Surgery year, *n* (%)	2016	96 (20.6)	68 (20.8)	28 (20.0)	0.516
	2017	125 (26.8)	93 (28.4)	32 (22.9)	
	2018	138 (29.6)	91 (27.8)	47 (33.6)	
	2019	108 (23.1)	75 (22.9)	33 (23.6)	
Age in years, median (IQR)		51 (43–58)	51 (43–60)	49 (43–55)	0.031
Focality, *n* (%)	Single	372 (79.6)	286 (87.5)	86 (61.4)	<0.001
	Multifocal	95 (20.3)	41 (12.5)	54 (38.6)	
cT, *n* (%)	cT1	131 (28.1)	95 (29.1)	36 (25.7)	<0.001
	cT2	269 (57.6)	204 (62.4)	65 (46.4)	
	cT3	42 (9.0)	22 (6.7)	20 (14.3)	
	cT4	25 (5.4)	6 (1.8)	19 (13.6)	
cN, *n* (%)	cN0	243 (52.0)	187 (57.2)	56 (40.0)	0.001
	cN+	224 (48.0)	140 (42.8)	84 (60.0)	
Stage, *n* (%)	I	85 (18.2)	63 (19.3)	22 (15.7)	<0.001
	II	306 (65.5)	234 (71.6)	72 (51.4)	
	III	76 (16.3)	30 (9.2)	46 (32.9)	
Ki67%, median (IQR)		50 (35–78)	60 (40–80)	45 (30–70)	0.006
Grading, *n* (%)	1	59 (12.6)	45 (13.8)	14 (10.0)	0.384
	2	100 (21.4)	66 (20.2)	34 (24.3)	
	3	273 (58.5)	193 (59.0)	80 (57.2)	
	Missing	35 (7.5)	23 (7.0)	12 (8.5)	
Molecular subtype	HR+/HER2−	62 (13.3)	44 (13.5)	18 (12.9)	0.012
	HR+/HER2+	135 (28.9)	89 (27.2)	46 (32.9)	
	HR−/HER2+	126 (27.0)	79 (24.2)	47 (33.6)	
	Triple Negative	144 (30.8)	115 (35.2)	29 (20.7)	
Post-operative radiotherapy, *n* (%)		369 (79%)	301 (92.0)	68 (48.6)	<0.001
Adjuvant endocrine therapy, *n* (%)		201 (43.1)	138 (42.2)	63 (45.0)	0.576

Footnotes: BCS. breast-conserving surgery; HR, hormone receptor; IQR, interquartile range.

**Table 2:** Univariate and multivariate Cox regression analysis for recurrence-free survival.

		Univariate			Multivariate		
Variables		HR	95% CI	*p*-Value	HR	95% CI	*p*-Value
Surgery type	BCS	ref.			ref.		
	Mastectomy	2.48	1.27–4.86	0.008	2.08	1.05–4.11	0.035
Age		0.98	0.94–1.01	0.156	0.98	0.94–1.01	0.161
Focality	Single	ref.					
	Multifocal	1.97	0.96–4.05	0.064			
cT	cT1	ref.					
	cT2	2.13	0.80–5.66	0.128			
	cT3	2.76	0.74–10.28	0.130			
	cT4	4.44	1.19–16.56	0.026			
cN	cN0	ref.			ref.		
	cN+	3.31	1.55–7.10	0.002	3.02	1.40–6.51	0.005
Molecular subtype	HR+/HER2−	ref.					
	HR+/HER2+	0.56	0.17–1.85	0.344			
	HR−/HER2+	1.2	0.42–3.41	0.732			
	Triple Negative	0.96	0.33–2.75	0.935			
Radiotherapy	No	ref.					
	Yes	0.99	0.43–2.27	0.974			
Endocrine therapy	No	ref.			ref.		
	Yes	0.65	0.31–1.33	0.233	0.57	0.27–1.18	0.130

Footnotes: BCS. breast-conserving surgery; HR, hazard ratio; CI, confidence interval. Multivariable Cox analysis performed after a stepwise selection of variables starting from a saturated model.

**Table 3:** Univariate and multivariate Cox regression analysis for overall survival.

		Univariate			Multivariate		
Variables		HR	95% CI	*p*-Value	HR	95% CI	*p*-Value
Surgery type	BCS	ref.			ref.		
	Mastectomy	1.86	0.85–4.11	0.122	1.91	0.84–4.33	0.121
Age		1.00	0.97–1.04	0.938			
Focality	Single	ref.					
	Multifocal	1.54	0.64–3.68	0.334			
cT	cT1	ref.					
	cT2	1.26	0.45–3.53	0.663			
	cT3	3.16	0.91–10.91	0.069			
	cT4	2.06	0.40–10.64	0.387			
cN	cN0	ref.			ref.		
	cN+	5.93	2.04–17.29	0.001	6.16	2.09–18.17	0.001
Molecular subtype	HR+/HER2−	ref.			ref.		
	HR+/HER2+	0.45	0.09–2.25	0.333	0.39	0.08–1.97	0.257
	HR−/HER2+	1.14	0.29–4.41	0.849	0.79	0.20–3.08	0.732
	Triple Negative	1.75	0.49–6.20	0.386	1.97	0.55–7.01	0.295
Radiotherapy	No	ref.					
	Yes	1.05	0.39–2.79	0.924			
Endocrine therapy	No	ref.					
	Yes	0.51	0.21–1.23	0.133			

Footnotes: HR, hazard ratio; CI, confidence interval. Multivariable Cox analysis performed after a stepwise selection of variables starting from a saturated model.

## Data Availability

Dataset available on request from the authors. The raw data supporting the conclusions of this article will be made available by the authors upon request.
